# Determination of Stable Hydrogen Isotopic Composition and Isotope Enrichment Factor at Low Hydrogen Concentration

**DOI:** 10.1021/acs.analchem.3c03214

**Published:** 2023-10-25

**Authors:** Xiao Liu, Langping Wu, Steffen Kümmel, Matthias Gehre, Hans Hermann Richnow

**Affiliations:** † Department of Isotope Biogeochemistry, Helmholtz Centre for Environmental Research-UFZ,Permoserstraße 15, 04318 Leipzig, Germany; ‡ Ecometrix Incorporated, 6800 Campobello Road, Mississauga, ON L5N 2L8, Canada; § Isodetect GmbH, Deutscher Platz 5b, 04103 Leipzig, Germany

## Abstract

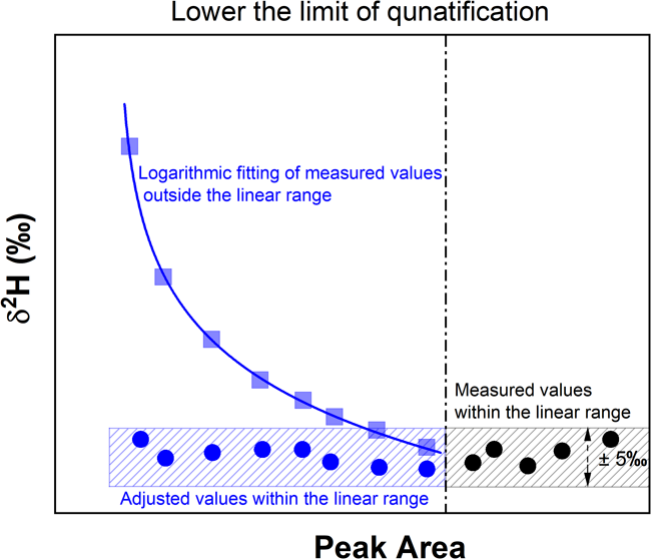

Determination of stable hydrogen isotopic compositions (δ^2^H) is currently challenged to achieve a high detection limit for reaching the linear range where δ^2^H values are independent of concentration. Therefore, it is difficult to assess precise δ^2^H values for calculating the hydrogen isotope enrichment factor (ε_H_) and for field application where the concentrations of contaminants are relatively low. In this study, a data treatment approach was developed to obtain accurate δ^2^H values below the linear range. The core concept was to use a logarithmic function to fit the δ^2^H values below the linear range and then adjust the δ^2^H values below the linear range into the linear range by using the fitted logarithmic equation. Moreover, the adjusted δ^2^H values were calibrated by using laboratory reference materials, e.g., *n*-alkanes. Tris(2-chloroethyl) phosphate (TCEP) and hexachlorocyclohexane (HCH) isomers were selected as examples of complex heteroatom-bearing compounds to develop the data treatment approach. This data treatment approach was then tested using δ^2^H values from a TCEP transformation experiment with OH radicals. Comparable δ^2^H values and ε_H_ between the low-concentration experiment and the reference experiment were obtained using the developed approach. Therefore, the developed data treatment approach enables a possibility of determining the hydrogen isotopic compositions of organic components in low concentrations. It is especially valuable for determining organic contaminants in environmental samples, which are usually present in low concentrations.

## Introduction

Compound-specific isotope analysis (CSIA) has been widely applied to characterize the fate of organic pollutants in the environment.^1−6^ Accordingly, CSIA techniques for carbon (δ^13^C), nitrogen (δ^15^N), chlorine (δ^37^Cl), sulfur (δ^34^S), and hydrogen isotopic compositions (δ^2^H) have been developed in the past.^7−12^ Combined with precise δ^2^H values, the CSIA of contaminants can provide useful information to distinguish among sources for isotope forensics. Furthermore, different reaction mechanisms can be characterized by interpreting not only the primary hydrogen isotope effects caused by the changing (cleavage or formation) of a hydrogen containing bond but also the secondary hydrogen isotope effects at chemical entities where hydrogen is not directly involved in the bond changing but is located in the vicinity of the changing bond. In summary, the δ^2^H values are often needed in the case of multielement isotope analysis, which can be used for a detailed characterization of transformation processes and for the source identification of contaminants.^13−15^

Direct pyrolytic conversion of organic compounds into H_2_ via a high-temperature conversion (HTC) unit at *T* > 1050 °C has allowed a significantly improved application of online IRMS techniques.^16,17^ However, applying this method to organic compounds containing heteroatoms (e.g., N, S, Cl) can lead to the formation of H-containing byproducts (e.g., HNC, H_2_S, HCl) which prevents the accurate, precise, and reliable determination of δ^2^H values.^18,19^ To overcome this problem, quantitative conversion of the H-containing byproducts to H_2_ is needed to circumvent possible isotope effects caused by incomplete conversion. Therefore, a chromium-based reactor system (Cr/HTC) has been recently developed to quantitatively scavenge heteroatoms by the formation of chromium salts and thus enable the quantitative conversion of all organic-bounded hydrogen atoms to H_2_, resulting in accurate δ^2^H values.^11,19,20^

The limit of quantification (LOQ) for ^2^H is relatively high compared to ^13^C analysis. The range where the isotopic composition of ^2^H is independent of the concentration is often referred to as the linear range. Determining the range of analyst concentrations for obtaining reliable ^2^H values is a challenge for CSIA of ^2^H isotope analysis, as its sensitivity is relatively low. One of the obstacles for the analysis of δ^2^H values is the high LOQ to reach the linear range where δ^2^H values become independent of the concentration applied to the GC-IRMS. The linear range of δ^2^H values using the Cr/HTC approach is generally independent of the compound being analyzed and is usually satisfactory at signal intensities (*m*/*z* 2) above 4000 mV, corresponding to approximately 80 nmol H injected on column.^12^ This requirement of high amounts of hydrogen to be injected on the column complicates the analyses of low-concentrated samples, such as those typically encountered with hydrophobic organic pollutants in contaminated field site samples. The enrichment of the analyte to achieve the LOQ not only is laborious and time-consuming but also enhances the risk of artificially altering the isotopic composition due to extraction procedures. Therefore, lowering the LOQ for online stable hydrogen isotope measurements is urgently needed.

The present study describes a new data treatment approach for the analysis of δ^2^H values and the determination of the hydrogen isotope enrichment factor (ε_H_) for samples containing hydrogen concentrations below the typical LOQ. The term low hydrogen concentration in the present study is used to describe samples with a hydrogen content below the LOQ for the analysis of δ^2^H values without adjusting the data using a logarithmic equation. For validating the data treatment approach, tris(2-chloroethyl) phosphate (TCEP) and different hexachlorocyclohexane (HCH) isomers, typical persistent organic pollutants, were selected as examples of polar and nonpolar heteroatom-bearing organic compounds, respectively. To demonstrate the applicability of the developed data treatment approach, TCEP degradation experiments under UV/H_2_O_2_ were conducted at concentrations below the LOQ and the corresponding ε_H_ values were calculated.

## Experimental Section

### Chemicals

Tris(2-chloroethyl) phosphate (TCEP, 97% purity) and α-, γ-, and δ-hexachlorocyclohexane (99% purity) were purchased from Sigma-Aldrich (Germany). International reference materials USGS69 (Reston Stable Isotope Laboratory, U.S. Geological Survey, Reston, VA) as well as newly developed C_12_, C_14_, C_15_, and C_17_*n*-alkane laboratory reference materials (≥99% pure) were used for calibration and normalization along the VSMOW isotope scale. The standard solution of TCEP and HCH isomers was prepared in acetone (ROTISOLV ≥ 99.9%, UV/IR-Grade). The standard solution of *n*-alkanes was prepared as a mixture of C_14_, C_15_, C_16_, and C_17_ in acetone (5 μL of each alkane in 1 mL of acetone).

### Concentration Analysis of TCEP

An Agilent 6890 series gas chromatograph (Agilent Technologies, Palo Alto) equipped with a flame ionization detector (FID) was used to determine the TCEP concentration throughout the study. Samples were separated using an HP-5 column (30 m × 320 μm × 0.25 μm, Agilent) with a constant helium carrier gas flow of 1.5 mL min^–1^. The oven was first held at 60 °C for 2 min, then increased at 10 °C min^–1^ to 160 °C, at 5 °C min^–1^ to 220 °C, and at 15 °C min^–1^ to a final temperature of 280 °C, and held for 2 min. Each sample was measured using a split ratio of 50:1. The injector temperature was 195 °C.

### Hydrogen Isotope Analysis

An Agilent 7890A series gas chromatograph (Agilent Technologies, Palo Alto) was coupled via a GC-Isolink (Thermo Fisher Scientific, Germany) equipped with a homemade Cr/HTC reactor maintained at 1200 °C and a ConFlo IV interface (Thermo Fisher Scientific, Germany) to a MAT 253 isotope ratio mass spectrometer (IRMS) system (Thermo Fisher Scientific, Germany). A detailed description of the Cr/HTC reactor design can be found elsewhere.^21^ For the analysis of TCEP, a DB-608 column (30 m × 0.32 mm × 0.5 μm, J&W collum, Agilent) was used employing the following oven temperature program: 60 °C for 2 min, then increased at 20 °C min^–1^ to 210 °C, at 1 °C min^–1^ to 220 °C, at 20 °C min^–1^ to a final temperature of 280 °C, and held for 5 min. Sample aliquots (1–3 μL) were injected into a split/splitless injector at 195 °C. For the analysis of HCH, a ZB-1 column (60 m × 0.32 mm × 1 μm; Phenomenex, Germany) was used employing the following oven temperature program: 40 °C for 5 min, then increased at 10 °C min^–1^ to 175 °C, at 2 °C min^–1^ to 200 °C with a hold of 10 min, and at 15 °C min^–1^ to a final temperature of 300 °C, followed by a hold for 2 min. Sample aliquots (1–3 μL) were injected into a split/splitless injector at 250 °C. All samples were analyzed in triplicate using a split ratio of 1:5.

Hydrogen isotopic compositions were reported as δ notations in parts per thousand (‰) relative to the international standard scale (Vienna Standard Mean Ocean Water (VSMOW)) according to eq 1:
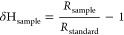
1where *R*_sample_ and *R*_standard_ are the ^2^H/^1^H ratios of the sample and the standard, respectively.

The H^3+^ factor was measured before and after a series of sample analyses and ranged over the course of all analyses from 10.24 to 10.29 ppm/nA in a linear range of 1 to 10 V. All peaks were integrated employing the same method. Peak integration was based on the *m*/*z* 2 signal. Start and end of the peak was identified by a slope of 2 and 4 mV/s, respectively.

### Normalization of Hydrogen Isotopic Compositions

The accurate assessment of δ^2^H values requires a two-point calibration employing at least two isotopically characterized reference materials (RMs) with contrasting isotopic compositions to (i) anchor the isotopic scale and (ii) compensate for differences in the response of the instrument.^22^ However, a major hindrance for a routine application of the online stable hydrogen isotope measurement techniques is the lack of suitable GC amenable organic isotope RMs. Recently, new organic RMs have been developed specifically for hydrogen isotope analysis, for example, the *n*-hexadecanes including USGS67, USGS68, and USGS69.^22^ Normalization of raw δ^2^H values on the VSMOW scale was achieved by applying a two-point calibration approach using the laboratory RMs *n*-tetradecane (C_14_, δ^2^H = −229 ± 2‰)^12^ and *n*-hexadecane (USGS69, C_16_, δ^2^H = 381 ± 3‰).^22^ In addition, *n*-pentadecane (C_15_, δ^2^H = −67 ± 2‰) and *n*-heptadecane (C_17_, δ^2^H = −72 ± 2‰)^12^ were used for the validation of the calibration.

### Calculation of the Hydrogen Isotope Enrichment Factor

The hydrogen isotope enrichment factor (ε_H_) was determined using the logarithmic form of the Rayleigh equation^23^ by plotting ln(*C*_t_/*C*_0_) versus ln[(δ_t_^2^H + 1)/(δ_0_^2^H + 1)]. The ε_H_ was obtained from the slope of the linear regression according to eq 2
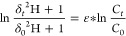
2where *C_t_* and *C*_0_ represent the concentration of the target compound at time *t* and at the initial time, respectively, and δ*_t_*^2^H and δ_0_^2^H represent the hydrogen isotopic composition of the target compound at time *t* and at the initial time, respectively.

### TCEP Transformation Experiment

**Scheme 1:**
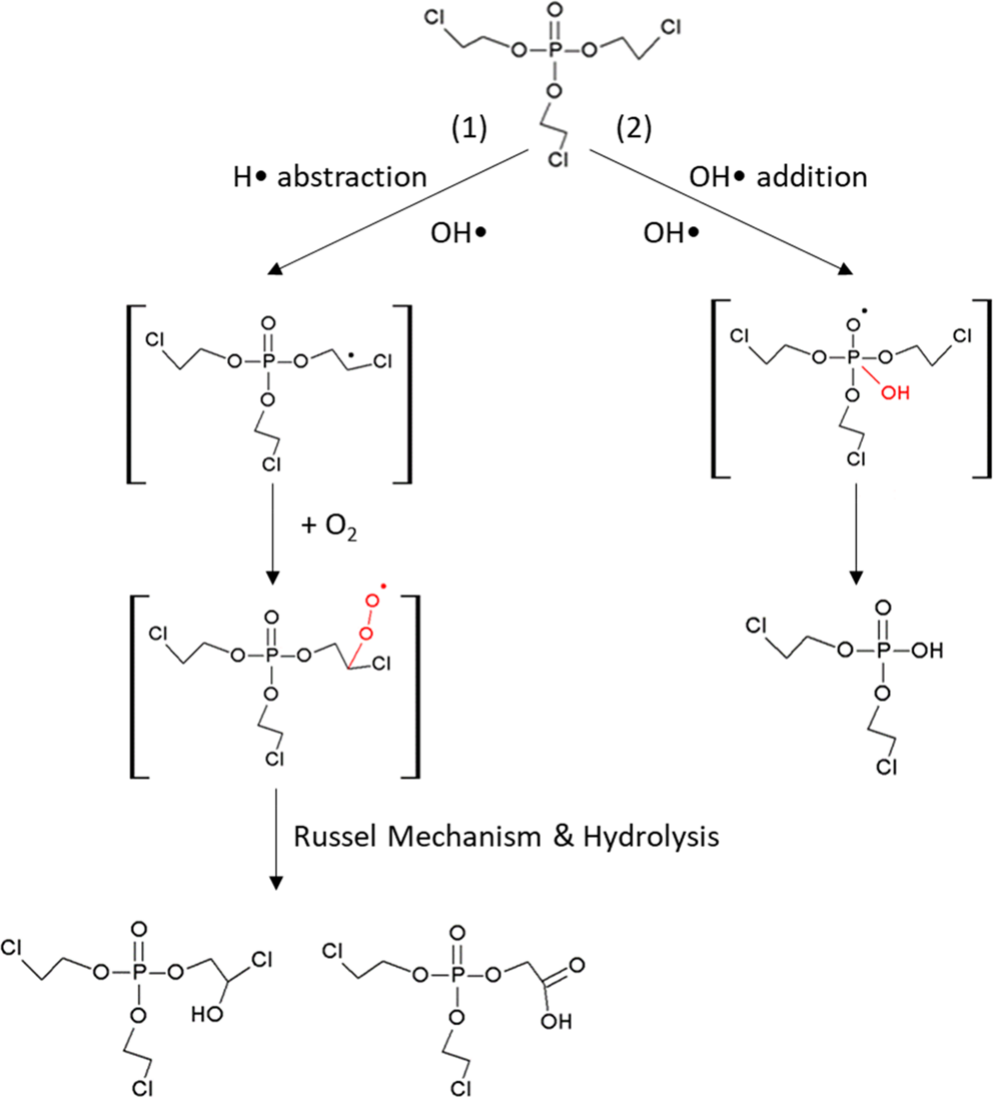
Proposed Transformation Mechanisms of the OH radical Reaction with TCEP It illustrates that the first step reaction of TCEP may simultaneously occur via two different pathways: (1) involves hydrogen abstraction by OH radicals, followed by oxygen addition and then undergo Russell Mechanism and hydrolysis and (2) involves OH radical addition to the central phosphorus atom, followed by the elimination of an ethyl-chlorine arm from the phosphoric center.

TCEP can be oxidized by OH radicals, which were formed by the irradiation of H_2_O_2_ with artificial sunlight. The reaction was conducted in a photochemical reactor system consisting of a 200 mL Pyrex cylindrical flask and a circulating water system. Irradiation was achieved using a 150 W xenon lamp as light source (Type L2175, wavelength: 185–2000 nm, Hamamatsu, Japan). A filter with a cutoff wavelength of 280 nm (Schott WG 280 long pass filter, 3.15 mm thick, Galvoptics Ltd., United Kingdom) was applied to provide an emission spectrum with wavelengths ≥280 nm which are typical for sunlight reaching the Earth’s surface. Experiments were conducted in phosphate buffer (100 mM, pH 7) at 20 °C, with an initial concentration of 1000 mg L^–1^ TCEP. 30% H_2_O_2_ (30%) was used to obtain an initial molar ratio of H_2_O_2_/TCEP of 50:1. Details of degradation experiments are reported elsewhere.^21^ The degradation pathways of TCEP oxidized by OH radicals are studied elsewhere^21^ and represented in Scheme 1, which shows that the first step of the reactions occurs simultaneously via two different pathways (hydrogen abstraction and OH radical addition).^21^ The major pathway is hydrogen abstraction by OH radicals accompanied by a C–H bond cleavage, followed by an oxygen addition. The samples from the degradation experiment were analyzed twice. The first set of analyses was conducted to obtain δ^2^H values with peak areas above the LOQ without adjusting the data using a logarithmic equation, which is referred to as the reference experiment. The second set of analyses was conducted to obtain δ^2^H values with peak areas below the LOQ without adjusting the data using a logarithmic equation by diluting the samples prior to the analysis, which is referred to as the low-concentration experiment in this study.

## Results and Discussion

### Development of a Data Treatment Approach for the Analysis of δ^2^H Values at a Low-Concentration Level

The analysis of isotopic compositions of heteroatom-bearing compounds is more complicated compared to that of aliphatic and aromatic hydrocarbons. Heteroatom-bearing compounds give broad H_2_ peaks with tailings due to their poor gas chromatographic separation and limited thermostability, leading to earlier and partial pyrolytic decomposition to not only form H_2_ but also H-containing byproducts, which need to be converted to H_2_ for quantitative transformation. Thus, TCEP and HCH were selected as examples of chlorinated heteroatom-bearing compounds to develop and evaluate the concept of extending the LOQ.

#### Assessing the Dependency of δ^2^H Values on Concentration

**Figure 1:**
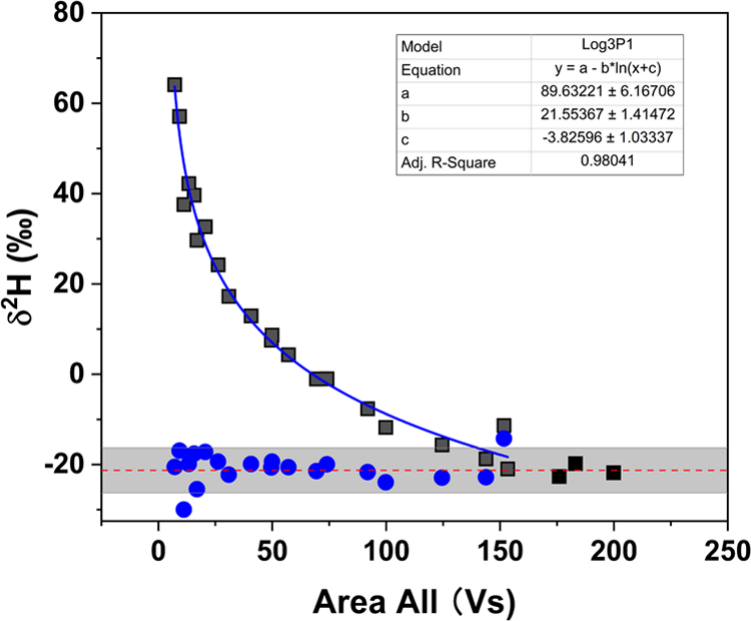
Hydrogen isotopic compositions of TCEP obtained by injecting different amounts of TCEP standard solution into the GC-Cr/HTC-IRMS (black squares). Adjustment of δ^2^H values below the LOQ (150 Vs) was done by recalculating the δ^2^H values (blue circles) based on the derived logarithmic equation (blue line). The red dash line represents the average δ^2^H value of TCEP (−21 ± 5‰, *n* = 24) within the linear range with uncertainty (gray bar), which was calculated using the adjusted δ^2^H values and the unadjusted ones above the LOQ.

**Figure 2:**
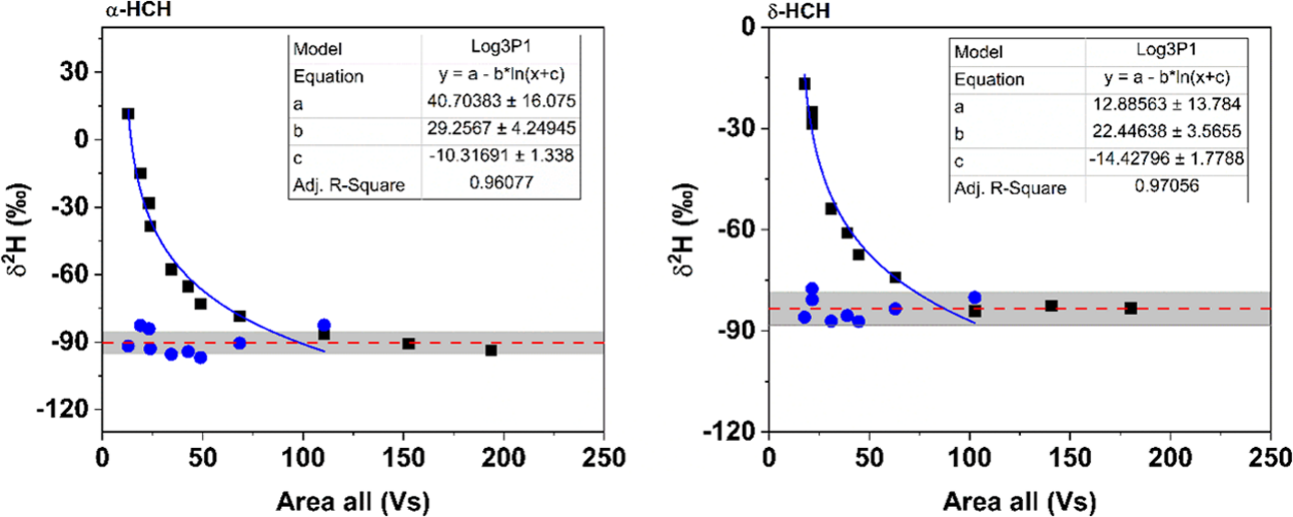
Hydrogen isotopic compositions of α- and δ-HCH obtained by injecting different amounts of HCH standard solution into the GC-Cr/HTC-IRMS (black squares). Adjustment of δ^2^H values below the LOQ (100–150 Vs) was done by recalculating the δ^2^H values (blue circles) based on the derived logarithmic equations (blue line), respectively. The red dash lines represent the average δ^2^H values of α-HCH (−90 ± 5‰, *n* = 11) and δ-HCH (−84 ± 5‰, *n* = 10), respectively, within the linear range with uncertainty (gray bar), which were calculated using the adjusted δ^2^H values and the unadjusted ones above the LOQ.

Measurements of different amount of TCEP standard solution demonstrated a dependence of δ^2^H values on the TCEP concentration when the resulted peak area was smaller than 150 Vs, which corresponds to 260 nmol H injected on the column (Figure 1). In contrast, independence of the δ^2^H values from the TCEP concentration was observed when the TCEP signals were higher than 150 Vs (Figure 1). In the current study, the peak area (Vs) instead of the peak amplitude (mV) of *m*/*z* 2 was used for interpretation since the peak area is directly related to the concentration, whereas the amplitude *m*/*z* 2 is affected not only by the concentration but also by the peak shape and thus influenced by the chromatography effect and peak tailing. Additionally, the dependence of δ^2^H values on the concentration of different HCH isomers was assessed, resulting in a similar trend with that for TCEP. The δ^2^H values of the tested HCH isomers became independent of the concentration when an area of 100–150 Vs was reached, which corresponds to at least 315 nmol H injected on the column (Figure 2). In contrast, the δ^2^H values of the investigated *n*-alkanes (C_12_, C_14_, C_15_, C_16_, and C_17_) showed an independence of the concentration when the respective peak area was higher than 35 Vs, which corresponds to a signal intensity (*m*/*z* 2) of approximately 4000 mV and corresponds to 80 nmol H injected on the column as previously reported.^12^ The LOQs of the *n*-alkanes are much lower than those of TCEP and HCH. The variation of LOQs could be affected not only by the peak shape of the chromatogram but also by the molecular composition and the chemical structure of the individual compound. *n*-Alkanes only contain carbon and hydrogen atoms, whereas TCEP and HCH contain a phosphate group and chlorinated carbon moieties, which are likely more difficult to be converted quantitatively via the Cr/HTC approach.

#### Adjustment of δ^2^H Values in the Nonlinear Range

For the δ^2^H values of TCEP in the nonlinear range, the correlation between peak areas and the corresponding δ^2^H values could be described by a logarithmic function (δ^2^H values = 89.6 – 21.6 × ln(peak area – 3.8)) with a significant correlation of *R*^2^ = 0.98 (Figure 1). Thus, δ^2^H values of TCEP fell out of the linear range (peak area <150 Vs) could be adjusted to the linear range by recalculating the δ^2^H values using the derived logarithmic equation (Figure 1). Based on this approach, the δ^2^H values in the nonlinear range were adjusted to a peak area of 150 Vs. All injected TCEP standards, including the adjusted δ^2^H values in the nonlinear range and the ones obtained in the linear range (without adjustment), resulted in a mean δ^2^H value of −21 ± 5‰ (*n* = 24) (Figure 1). Hence, the LOQ could be lowered by a factor of 15 since the linear range could be expanded to peaks with an area of 10 Vs. To further validate the above data treatment approach, the method was applied to HCH isomers, resulting in a comparable result as that observed for TCEP (Figure 2). The LOQ could be lowered by a factor of 8–9 for the studied HCH isomers. Consequently, the results indicate that the developed approach can be used to adjust δ^2^H values obtained in the nonlinear range to be comparable with those obtained in the linear range. However, the extent of the reduction of the LOQ is affected by the molecular composition and the chemical structure of the individual compound.

It is worth noting that the derived logarithmic equation is highly related to not only the physiochemical properties of the measured compounds but also the conditions of the conversion reactor of the IRMS. In order to obtain a reliable logarithmic equation and to determine the influence of the approach on the LOQ, tests with standards are required before every hydrogen isotope analysis.

#### Normalization of δ^2^H Values

**Scheme 2:**
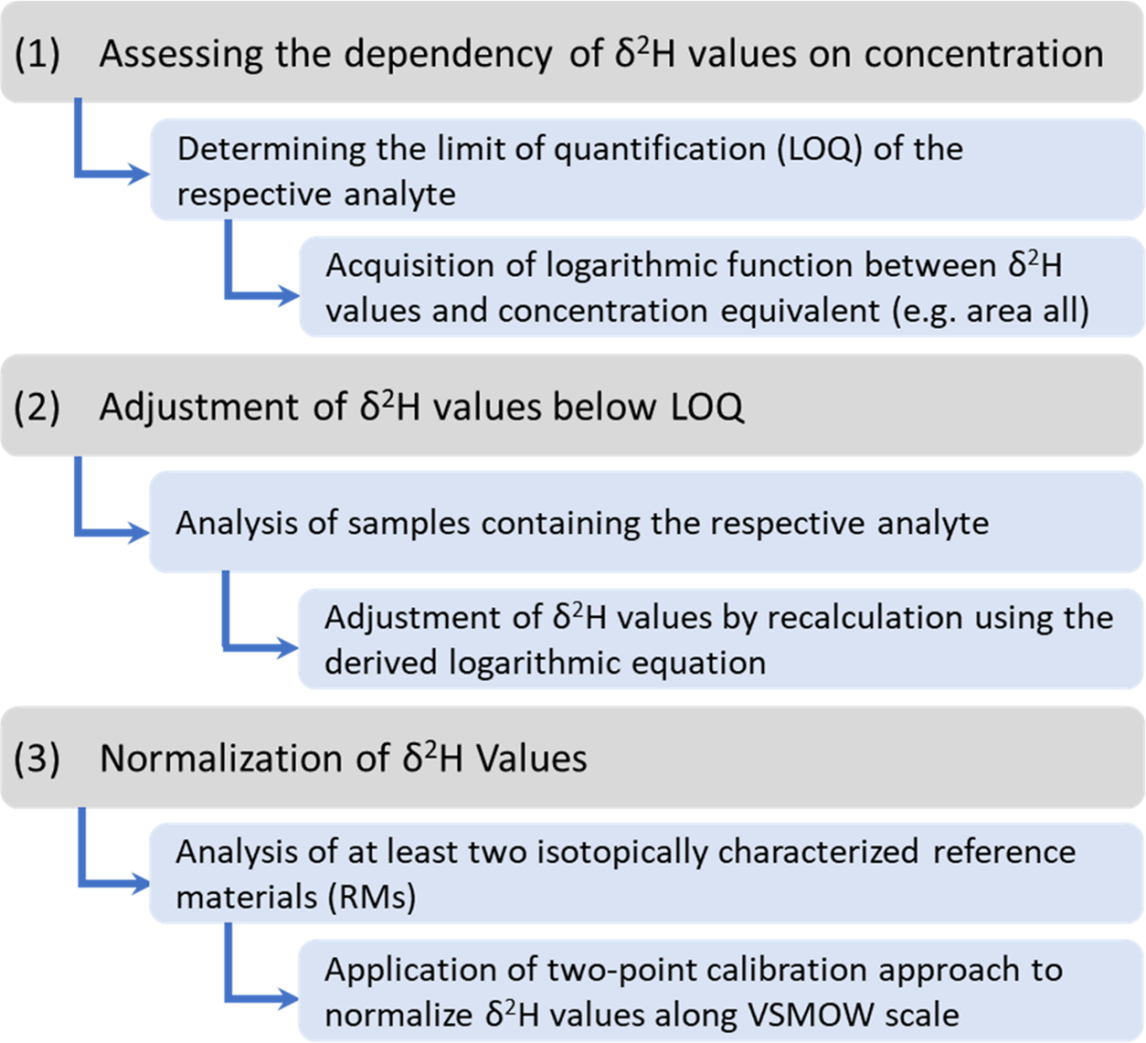
Proposed Data Treatment Approach for the Analysis of δ^2^H Values at Low-Concentration Levels

After the adjustment of the δ^2^H values in the nonlinear range, all of the adjusted as well as the unadjusted δ^2^H values were normalized and validated by applying a two-point calibration approach using the laboratory RMs C_14_, C_15_, C_17_,^12^ and the international RM USGS69.^22^ Thus, all GC-IRMS derived data were uniformly normalized to compensate for any scale compression of the mass spectrometer and to obtain corrected δ^2^H values along the VSMOW scale. The concentrations of the *n*-alkanes were adjusted to ensure that the peak areas were higher than 35 Vs (Table 1) to guarantee that the determined δ^2^H values of *n*-alkanes are in their linear range. A summary of the above data treatment approach is shown in Scheme 2.

**Table 1:** Area All, Ampl.2, and Hydrogen Isotopic Compositions of *n*-Alkanes Measured by GC-IRMS and Reference Hydrogen Isotopic Compositions of *n*-Alkanes Measured by EA-IRMS

*n*-alkanes	area all (Vs)	ampl. two (mV)	δ^2^H (‰) (GC-IRMS)^a^	δ^2^H (‰) (EA-IRMS)^b^
*n*-tetradecane (C_14_)	35.88	4130	–203 ± 2	–229 ± 2^a^
*n*-pentadecane (C_15_)	36.21	4413	–54 ± 1	–67 ± 2^a^
*n*-hexadecane (C_16_) (USGS69)	37.65	4734	375 ± 1	381.4 ± 3^b^
*n*-heptadecane (C_17_)	36.61	4580	–51 ± 1	–72 ± 2^a^

a The values are from Renpenning et al.^24^

b The values are from Schimmelmann et al.^25^

GC-IRMS: gas chromatography–isotope ratio mass spectrometry.

EA-IRMS: elemental analyzer–isotope ratio mass spectrometry.

### Case Study: Stable Hydrogen Isotope Enrichment Factor during the Transformation of TCEP

**Figure 3:**
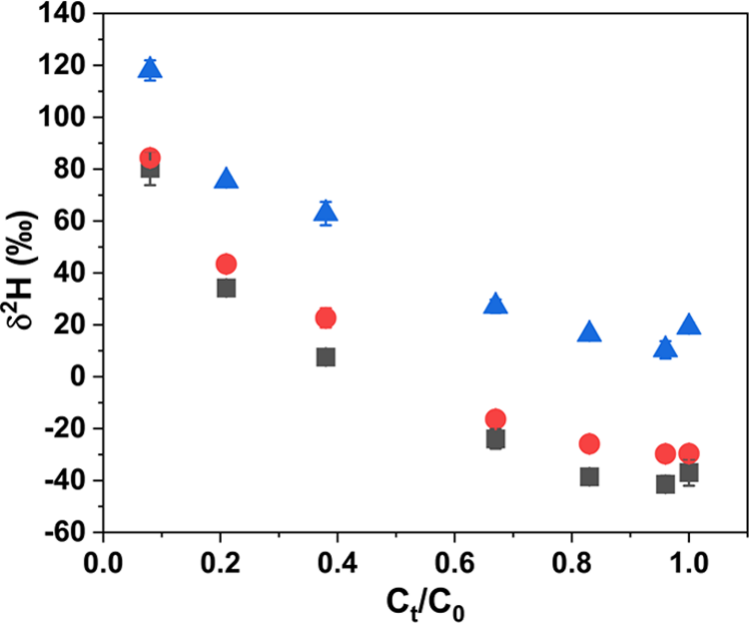
Changes of the hydrogen isotopic composition versus the remaining fraction of TCEP during the transformation experiment with OH radicals. The δ^2^H values obtained from the reference experiment are presented after normalization (gray squares). The δ^2^H values obtained from the low-concentration experiment are displayed as raw δ^2^H values (blue triangles) and adjusted δ^2^H values after recalculation and normalization (red circles), respectively.

To prove the developed data treatment approach, TCEP transformation experiments with OH radicals were conducted, and the stable hydrogen isotopic composition of the remaining TCEP fraction was analyzed. Thereby, each sample was analyzed two times to obtain two data sets: (i) one data set was obtained with peak areas above the LOQ (reference experiment), and (ii) one data set was obtained with peak areas below the LOQ by diluting the samples prior analysis (low-concentration experiment). The δ^2^H values obtained from the reference experiment were only normalized by applying the laboratory RMs, which are referred to as the reference δ^2^H values (gray squares in Figure 3). The δ^2^H values obtained from the low-concentration experiment were first adjusted using the derived logarithmic equation (Figure 1) and then normalized by laboratory RMs, which are referred to as the adjusted δ^2^H values (red circles in Figure 3). Neither adjusted nor normalized δ^2^H values obtained from the low-concentration experiment are referred to as the raw δ^2^H values (blue triangles in Figure 3). Remarkably, the raw δ^2^H values from the low-concentration experiment deviated significantly from the reference δ^2^H values (differences from 38 to 56‰), while the adjusted δ^2^H values were close to the reference δ^2^H values (differences from 4 to 15‰) (Table 2). Therefore, the application of the developed data treatment approach could improve the accuracy of the δ^2^H values by 80% compared to the raw δ^2^H values from the low-concentration experiment. Considering the typical analytical uncertainty of δ^2^H analysis (±5‰), the observed shifts between the adjusted and the reference δ^2^H values are acceptable.

**Table 2:** Hydrogen Isotopic Compositions and Isotope Enrichment Factor (ε_H_) for the Reference Experiment (Peak Areas above LOQ) and for the Low-Concentration Experiment (peak areas below LOQ) during the Transformation Experiment with OH Radicals

		reference experiment		low-concentration experiment					
		peak areas ≥150 Vs (above LOQ)		peak areas <150 Vs (below LOQ)					
		reference δ^2^H (‰)		raw δ^2^H (‰)			adjusted δ^2^H (‰)		
time (h)	*C_t_*/*C*_0_	ave.	stdv	ave.	stdv	diff.^a^	ave.	stdv	diff.^b^
0	1.00	–37	5	19	2	**56.**	–30	5	**7**
2	0.96	–41	3	10	4	**52**	–30	1	**12**
4	0.83	–39	1	16	1	**55**	–26	5	**13**
9	0.67	–24	4	27	3	**51**	–16	3	**8**
23	0.38	8	1	63	5	**55**	23	4	**15**
34	0.21	34	2	75	2	**41**	43	2	**9**
48.5	0.08	80	7	118	4	**8**	84	3	**4**
ε_H_ ± 95%CI (‰)		–47 ± 2		–39 ± 3			–45 ± 2		

a The difference of δ^2^H values between the unadjusted δ^2^H values and the reference δ^2^H values.

b The difference of δ^2^H values between the adjusted δ^2^H values and the reference δ^2^H values.

In the next step, the δ^2^H values were used to calculate the hydrogen isotope enrichment factor (ε_H_, Table 1). Notably, the ε_H_ obtained by using the adjusted δ^2^H values were comparable with the ε_H_ obtained with the reference δ^2^H values (−45 ± 2‰ Vs –47 ± 2‰). In contrast, a difference of 8‰ was observed between the ε_H_ obtained by using the raw δ^2^H values and the ε_H_ obtained with the reference δ^2^H values (−39 ± 3‰ Vs −47 ± 2‰). Thus, the developed data treatment approach could be used to analyze δ^2^H values and the corresponding isotope enrichment factors of low-concentration samples, which is especially important for environmental samples.

## Conclusions

A data treatment approach was developed to calculate δ^2^H values and ε_H_ when sample concentrations (and thus hydrogen amounts) are below the LOQ. The core concept is based on an adjustment of the δ^2^H values, followed by a normalization along the VSMOW scale. The results of the tested model polar (TCEP) and nonpolar (HCH isomers) compounds illustrate that the developed approach could be applied to compounds with different physiochemical properties. By applying this approach to the investigated heteroatom-bearing compounds, the LOQ could be lowered by a factor of 15 for TCEP and 8–9 for HCH isomers, indicating that the extent of the LOQ reduction is influenced by the molecular composition and the chemical structure of the individual compound. To obtain a reliable logarithmic equation and to determine the particular influence of the approach on the LOQ for a given compound, tests with its analytical standard are required prior to any hydrogen isotope analysis of a sample. Moreover, the developed data treatment approach was successfully applied to determine the ε_H_ associated with the transformation of TCEP via OH radical oxidation, resulting in more accurate δ^2^H and ε_H_ values. Consequently, the developed data treatment approach enables an extended linear range for the analysis of the isotopic compositions of organic components in environmental samples that are often present at low concentrations.
